# PReS-FINAL-2147: Immunological consequences of biologicals in juvenile idiopathic arthritis

**DOI:** 10.1186/1546-0096-11-S2-P159

**Published:** 2013-12-05

**Authors:** JF Swart, S De Roock, NM Wulffraat

**Affiliations:** 1 Pediatric Immunology, UMC Utrecht, Utrecht, Netherlands

## Introduction

Since biologicals antagonize cytokines or receptors involved in the immune system, one could fear that their (long-term) use might affect the quality of the immune system, leading to a defective defense mechanism against infections and tumors, an insufficient response to vaccinations, or a flawed immunoregulation resulting in autoimmunity or autoinflammation. Finally, a biological agent itself can be handled as an antigen by the immune system, producing antibodies against the biological.

## Objectives

To provide a current overview of adverse events for each biological agent used in JIA calculated as incidence per 100 patientyears for the following categories: serious infections, tuberculosis, malignancies, response to vaccination, new-onset autoimmune diseases and development of anti-drug antibodies.

## Methods

This study is an in-depth review resulting from a meta-analysis of all the available data on pubmed concerning the (immunological) side effects of biological response modifiers used to treat juvenile arthritis.

## Results

The immunological consequences of the long-term use of biologicals differ per agent and are highly dependent on concomitant medication. Infection risk is mainly associated with JIA itself and moderate to high doses of steroids and not the use of a biological, although tocilizumab might be an exception to this rule. Malignancies do not seem to occur more in JIA patients using anti-TNF therapy, although the background rate of JIA patients might be higher than that of the general population. Generally, the immunogenicity of vaccines is good in JIA patients, but one should be cautious when administering new live attenuated vaccines in patients with high dose immunosuppressants, including biologicals. There is an increased incidence of demyelinating diseases, IBD and development of mostly clinically irrelevant auto-immune antibodies in JIA patients on anti-TNF. The occurrence of uveitis does not seem to be increased in patients with etanercept or abatacept. Anti-drug antibody formation is seen in many patients with monoclonal antibodies, especially when they do not use concomitant MTX, but these antibodies are mostly not correlated to clinical events.

## Conclusion

There are large differences in side effects between various agents and there is a clear need for an international and standardized collection of post-marketing surveillance data of biologicals in the vulnerable group of JIA patients. Such an international pharmacovigilance database, called Pharmachild, has now been started.

## Disclosure of interest

None declared.

